# Factors associated with low birth weight in low-income populations in the Western Balkans: insights from the multiple indicator cluster survey

**DOI:** 10.3389/fpubh.2024.1394060

**Published:** 2024-12-10

**Authors:** Dragana Grbic, Zorica Terzic Supic, Jovana Todorovic, Dejan Nesic, Svetlana Karic, Aleksandar Jurisic, Sanja Kocic, Zoran Bukumiric, Andja Cirkovic, Svetlana Jankovic

**Affiliations:** ^1^Medical Faculty Belgrade, Gynecology Obstetric University Clinic Narodni Front, Belgrade, Serbia; ^2^Institute of Social Medicine, Medical Faculty Belgrade, University of Belgrade, Belgrade, Serbia; ^3^Institute of Medical Physiology, Medical Faculty Belgrade, University of Belgrade, Belgrade, Serbia; ^4^Department of Studies for Preschool and Nursery Teachers, Academy of Professional Studies, Šabac, Serbia; ^5^Department of Social Medicine, Faculty of Medical Sciences, University of Kragujevac, Kragujevac, Serbia; ^6^Institute for Medical Statistics and Informatics, Medical Faculty Belgrade, University of Belgrade, Belgrade, Serbia

**Keywords:** low birth weight, socio-economic factors, illiteracy, antenatal care, wealth index

## Abstract

**Introduction:**

Low birth weight, defined as a birth weight below 2,500 g, represents a significant public health concern with a multifactorial risk dimension. Socio-demographic factors and individual characteristics of women and their social environment could influence low birth weight. This study aimed to analyze the association between the socio-demographic and reproductive characteristics of women living in low-income households and low birth weight in Serbia, Kosovo, and Montenegro.

**Methods:**

This study was conducted as secondary data analysis during the Multiple Indicator Cluster Survey – Round 6 in Serbia, Kosovo, and Montenegro. The household questionnaire and the individual questionnaire for women aged 15–49 were used as standard research instruments. We analyzed 1,019 women whose households belonged to the first (poorest) or second (poor) wealth index quintiles and who had given birth to a live child within the 2 years preceding the study. A multivariate logistic regression was applied with low birth weight in newborns as the outcome variable.

**Results:**

The univariate regression analysis showed that women with low birth weight newborns were significantly more likely to live in settlements mainly inhabited by Roma, reside in urban areas, marry or enter a union before age 18, have lower education levels, experience higher illiteracy rates, and receive antenatal care not provided by a medical doctor compared to women whose newborns weighed 2.5 kg or more. A multivariate logistic regression model with a low birth weight of newborns as an outcome variable showed the association between women’s illiteracy (OR: 1.741; 95% CI: 1.060–2.859) and antenatal care not provided by a medical doctor (OR: 2.735; 95% CI: 1.229–6.087).

**Discussion:**

Illiteracy and limited access to medical doctor services during pregnancy were factors that increased the likelihood of low birth weight in newborns born to women living in low-income households in the selected Western Balkans populations. The cross-sectional design of this study does not allow the establishment of causal relationships among variables, but it can provide important evidence for future prevention strategies. Interventions are needed to enhance the education of women and to improve access to antenatal care across Serbia, Kosovo, and Montenegro.

## 1 Introduction

Low birth weight (LBW) is defined as a newborn weighing less than 2,500 g at birth and is a significant public health indicator reflecting maternal health, nutrition, socio-economic status, and healthcare access (1, 2). It is also a major public health concern with a multifactorial risk dimension (1).

Low birth weight newborns are 20 times more likely to die compared to newborns with a birth weight higher than 2,500 g (3). Immediate causes of LBW are premature birth (less than 37th gestational week), intrauterine fetal growth restriction, or both causes together (4, 5). Prematurity is the leading cause of death in children under 5 years of age in the world (6). Social risks for LBW are related to social inequalities (7, 8), ethnic and racial discrimination (9, 10), violence (11), and the low level of gross domestic product (GDP). The association between LBW and low household income was evident in national health surveys, which indicate that limited economic resources, material deprivation, and poor living conditions contribute significantly to this issue. In these population surveys, a wealth index was used to assess the socio-economic status of women and examine its association with LBW (7, 12–14).

Individual maternal characteristics that can increase the risk for LBW are constitutional attributes (short stature) (15), adolescent age (16) and age older than 35 years (17), lack of folic acid intake (18), stunting (19), obesity (20), health risk behavior such as smoking (21), and alcohol usage (22), and depression (23). Preterm birth and intrauterine growth restriction are associated with some maternal morbidities (hypertension and preeclampsia) and specific antenatal and obstetric conditions (genital infection and placental disorders) (24–26). Lack of maternal capacities, such as illiteracy, poor education (27), and single living (unmarried or without a partner) (28), can hinder a mother’s ability to meet her needs during the antenatal period, potentially leading to adverse pregnancy outcomes.

The LBW risk factors should be reviewed as part of standardized antenatal care, as recommended in the “Standards of Care for Women’s Health in Europe, Obstetric and Neonatal Services” by the European Board and College of Obstetrics and Gynecology (EBCOG) (29). These standards recommended that all women should have access to antenatal care, with risk factors identified and assessed to ensure focused care during pregnancy, all within an individualized care plan by the end of the 12th week of pregnancy (29). The EBCOG suggests that organizing standardized antenatal care for all women is a public health priority and a professional responsibility for obstetricians and their teams. The studies highlighted significant inequalities in access to antenatal care across European countries, linked to women’s low socio-economic status, limited education, and social deprivation (30–32). These factors play a substantial role in maternal morbidity and mortality, as well as fetal and neonatal outcomes.

According to the WHO and UNICEF estimates, 15–20% of live births per year in the world are children born with LBW (33). It is also estimated that 95% of the 20 million children with LBW are born in countries with low and middle socio-economic development (34). The percentage of live births with LBW in 2020 ranged from 4.0 to 12.9% among EU countries, with an average of 7% (33). The highest percentage of live births with LBW was in Turkey (12.9%), followed by Bulgaria (11.4%) and Greece (11.4%) (33). As part of Southeast Europe (SEE), the majority of the Western Balkans countries (Montenegro 6.2%, Serbia 6.2%, Albania 6.0%, Bosnia and Herzegovina 5.2%, and Croatia 5.0%) had estimated LBW prevalence that is lower than the European average, with the exception of North Macedonia 8.3% (33). The data on LBW in Kosovo were not presented (33).

The Western Balkans is a part of SEE, geographically, but geopolitically refers to six economies—Albania, Bosnia and Herzegovina, Kosovo, Montenegro, North Macedonia, and Serbia—that are not members of the European Union (35). The current population of the Western Balkans is approximately 17.6 million. This population is defined by a specific socio-political structure, shaped by demographic changes and characterized by its multinational and multi-confessional communities, a legacy of both historic and ongoing migrations (36). The social systems in the Western Balkans countries were once similar, but the past 20 years of transition have created differences that have impacted the health and healthcare of their populations. These differences, along with the efficiency of the public health system, can be linked to the socio-economic characteristics of each country, such as GDP *per capita* and ongoing political instability (37). A recent World Bank poverty consumption analysis in Kosovo for 2017 revealed that 18% of the population in Kosovo lives below the poverty line (estimated at 1.85 euros per day), while 5.1% lives below the extreme poverty line (estimated at 1.31 euros per day) (38). In the Serbian population, the at-risk-of-poverty rate decreased from 24.3 to 20.0% in the period from 2018 to 2022, but still Serbia is among Europe’s top 10 least equal countries, in terms of income (39, 40). In the same period in Montenegro, the at-risk-of-poverty rate was reduced from 23.8 to 20.3% (41). The countries of the Western Balkans are classified in the low-level development group of EU nations, characterized by middle-low incomes and a need to enhance their social and healthcare systems (42). The EU study, which had monitored the European Pillar of Social Rights in the Western Balkans countries since 2018, concluded that these countries made some progress in the necessary legal framework and social indicators, but welfare and labor market outcomes have remained generally weak, lagging behind EU standards (43). The OECD study illustrated a multi-dimensional review of the Western Balkans (35). It shows that GDP *per capita* in the Western Balkans is more than twice lower, the unemployment rate is three to four times higher, and education outcomes are below the average of OECD countries. Those poor economic outcomes have led to the lack of social protection and large inequalities between sub-regions and subpopulation groups. The social status of women in the Western Balkans is marked by low participation in paid work, primarily due to their full-time commitments to household responsibilities and caregiving for children and older adults. Cultural norms often limit their engagement in shared household earnings, contributing to increased economic dependency (35). This insufficient progress in welfare and regional social and economic inequalities underscores challenges within the healthcare system, encompassing issues such as healthcare coverage and investment in public health infrastructure, which directly correlates with the level of LBW prevalence in the Western Balkans (43, 44).

Health inequalities in the Western Balkans were also recognized and explored. The Multiple Indicator Cluster Survey (MICS), established by the UNICEF in 1996, collects data on the status and wellbeing of children, adolescents, and their families and serves to assess and monitor the needs of children in the Western Balkans (45). This survey is conducted every 5 years. The values of MICS indicators—related to housing characteristics (e.g., residence and wealth index), maternal health, child health, nutrition, and development—have provided crucial benchmarks for guiding preventive activities and assessing progress over the years, both in the general population and vulnerable subpopulation groups (45, 46). The last MICS (round 6) evidenced high LBW rates in the Roma population as a vulnerable group in the Western Balkans (Serbia – 11.0%, Kosovo – 8.7%, and Montenegro – 13.5%) (47–49). Other studies showed that Roma women have poor sexual and reproductive health (50, 51), do not use modern contraceptive methods (52), and are not in a position to decide about their healthcare and pregnancy (52). The study on access to maternal health and midwifery for vulnerable groups in the EU identified that women in the Roma community face significant barriers to accessing maternity care. They experience discrimination based on ethnicity, low economic status, place of residence, and language, leading to the poorest birth outcomes and maternal health (53).

To the best of our knowledge, there is currently no research on the factors contributing to LBW among newborns born to women with low socio-economic status in the Western Balkans. This study aimed to analyze the association between the socio-demographic and reproductive characteristics of women living in low-income households and low birth weight (LBW) in Serbia, Kosovo, and Montenegro.

## 2 Materials and methods

### 2.1 Population and sampling

This study was conducted as secondary data analysis during the MICS 6 (45). MICS6 was performed in the population of Montenegro in 2018 (49), Serbia in 2019 (47), and Kosovo in 2019/20 (48). The MICS 6 methodology, structured as a cross-sectional household study, provided a standardized protocol for all three authorities to follow. The MICS 6 datasets were accessed with the approval of the MICS team.

The two-stage stratified cluster sampling methodology for the urban/rural areas and the sub-region or districts was applied in the MICS 6. The representative household samples were drawn from the National Census of Population and Housing across all three populations. This included: (a) a household sample representing the general population and (b) a household sample from settlements inhabited by vulnerable minority groups, such as the Roma (47–49, 54).

The criterion for random household sampling was that at least one child under five had to be a household member. Surveys were conducted in respondents’ homes by trained interviewers, following the MICS research methodology (54), and with prior informed consent. Participation in MICS 6 was voluntary and anonymous for individuals present in the household during the survey who accepted to respond to the questionnaire. Detailed methodology is presented in the MICS 6 reports for Serbia, Kosovo, and Montenegro (47–49).

The MICS6 sample included 15,287 women aged 15–49, distributed as follows: Serbia – 3,740 women from the general population and 1,790 from Roma settlements; Kosovo – 5,275 women from the general population and 1,493 from Roma settlements; and Montenegro – 2,276 women from the general population and 713 from Roma settlements. We selected 1,019 women whose households belonged to the first (poorest) or second (poor) wealth index quintiles and who had given birth to a live child within the 2 years preceding the study. The collected data on household ownership of selected assets, dwelling characteristics, type of water access, and toilet and sanitation facilities were developed and standardized under the MICS research methodology to calculate the wealth index (54).

Exclusion criteria for the study were: households in the third (middle), fourth (rich), or fifth (richest) wealth index quintiles; women who had not given birth to a live child during the specified period; births that occurred outside a healthcare facility; and women who were unable to recall the birth weight.

### 2.2 Survey instrument

The household questionnaire and the individual questionnaire for women aged 15–49 were standard research instruments used in the MICS6 survey. We analyzed data from the following modules: household information panel, household characteristics, women’s information panel, women’s background, fertility, desire for the last birth, maternal and newborn health, victimization, and marital union.

We analyzed 19 variables. Women’s socio-demographic characteristics were described using 10 variables: the territory of woman’s origin (Serbia, Kosovo, and Montenegro), residence area (urban, rural), age, age at the first marriage/union (less than 18 years, 18 years, and more), marital status (married or with partner and no), literacy (“yes” if woman who had attended any type of preschool preparation program or school or could read a sentence, and “no” if she could not read a part or a whole sentence), education level (none, primary, secondary, and higher education), justification of domestic violence (category “yes” when woman justified husband/partner’s beating if she went out without telling him or/and if she neglected the children or/and if she argued with him or/and if she refused sex with him or/and if she burned the food or/and she justified his beating for any of these reasons) and positive feeling of discrimination (category “yes” if she felt discriminated because of her ethnic status or/and immigration origin or/and sex or/and sexual orientation or/and age or/and religion/belief or/and disability or/and she felt discriminated of any other reason). The variable “ethnicity” was determined based on the sample origin. Women were categorized as “non-Roma” if they were drawn from the general population sample and as “Roma” if they were from minority settlements (54).

Reproductive characteristics of women and the period of antenatal care and delivery were described by nine variables: parity (one, two, or three and more live births), the experience of the child’s death (yes, no), desire for last birth (wanted to get pregnant with the last child or not), antenatal care provided by a medical doctor (yes, no), number of antenatal care visits (less than eight; or eight and more) (55), pregnancy gestation at first antenatal care visit (up to 12 gestation weeks, over the 12 gestation weeks), place of delivery (in a hospital and not in a hospital), delivery assistance by a medical doctor (yes, no), and newborn birth weight (categorized as newborn birth weight < 2.5 kg and newborn birth weight ≥ 2.5 kg). The WHO antenatal care guideline served as a reference for the recommended number of visits, the gestational age for the initial antenatal care visit, and the threshold for low birth weight in newborns (55).

### 2.3 Statistical analysis

Categorical data were presented as absolute counts and percentages, while numerical data following a normal distribution were summarized using the arithmetic mean and standard deviation. Normality was assessed using both mathematical methods (Shapiro–Wilk and Kolmogorov–Smirnov tests, skewness and kurtosis, and the coefficient of variation) and graphical methods (histograms and box plots). In this study, we analyzed the differences between women who reported having a newborn weighing less than 2.5 kg (categorized as LBW) and those who reported having a newborn weighing 2.5 kg or more. The multicollinearity test was conducted. Student’s *t*-test or the Mann–Whitney test was used for numerical variables, depending on the data distribution. The chi-square test and Fisher’s exact test were used for categorical variables. Variables showing statistical significance (*p* ≤ 0.05) were included in the multivariate logistic regression, with LBW newborns born to women as the outcome variable. The variable “literacy” was included in the multivariate logistic regression as a more crucial measure of a woman’s educational capacity over “education level” for her health and wellbeing (56). The Enter method was applied, and the odds ratio (OR), 95% confidence interval (CI), and *p*-value were reported. Statistical significance was set at a *p*-value of ≤0.05. The analysis was performed using IBM SPSS Statistics for Windows, Version 21.0 (released in 2012), by IBM Corp., Armonk, NY, United States.

## 3 Results

The study included a sample of 1,019 women from low-income households, with 37.5% from Serbia, 38% from Kosovo, and 24.5% from Montenegro. Women with LBW newborns were significantly more likely to live in settlements mainly inhabited by Roma, reside in urban areas, marry or enter a union before age 18, have lower education levels, experience higher illiteracy rates, and receive antenatal care not provided by a medical doctor compared to women whose newborns weighed 2.5 kg or more (Table 1).

**Table 1:** Distribution of women’s characteristics by the birth weight of their newborns.

Women’s characteristics		Birth weight		
	Total, *N* (%)	<2.5 kg, *N* (%)	≥2.5 kg, *N* (%)	
	1,019 (100.0)	87 (8.5)	932 (91.5)	*p* ^a^
Territory of origin				
Serbia	382 (37.5)	29 (33.3)	353 (37.9)	0.453^b^
Kosovo	387 (38.0)	32 (36.8)	355 (38.1)	
Montenegro	250 (24.5)	26 (29.9)	224 (24.0)	
Ethnicity				
Non-Roma	606 (59.5)	41 (47.1)	565 (60.6)	0.014^b^
Roma	413 (40.5)	46 (52.9)	367 (39.4)	
Residence area				
Urban	420 (41.2)	45 (51.7)	375 (40.2)	0.037^b^
Rural	599 (58.8)	42 (48.3)	557 (59.8)	
Age of woman (mean ± SD)	27.8 ± 6.4	27.0 ± 7.1	27.9 ± 6.3	0.209^c^
Age at first marriage/union				
≥18 years	655 (64.5)	46 (52.9)	609 (65.6)	0.018^b^
<18 years	361 (35.5)	41 (47.1)	320 (34.4)	
Marital status				
Yes	969 (95.1)	84 (96.6)	885 (95.0)	0.794^d^
No	50 (4.9)	3 (3.4)	47 (5.0)	
Level of education				
None	403 (39.6)	50 (57.5)	353 (38.0)	<0.001^e^
Primary	259 (25.5)	17 (19.5)	242 (26.0)	
Secondary and high	355 (34.9)	20 (23.0)	335 (36.0)	
Literacy				
Yes	739 (72.7)	50 (58.1)	689 (74.1)	<0.001^b^
No	277 (27.3)	*36 (41.9)*	241 (25.9)	
Acceptance of domestic violence				
Yes	287 (28.2)	32 (36.8)	255 (27.4)	0.063^b^
No	731 (71.8)	55 (63.2)	676 (72.6)	
Felt discriminated				
Yes	108 (10.6)	14 (16.1)	94 (10.1)	0.082^b^
No	911 (98.4)	73 (83.9)	838 (89.9)	
Parity (Number of births)				
One	241 (23.7)	193 (23.1)	543 (32.0)	0.119^e^
Two	274 (26.9)	173 (20.7)	629 (37.0)	
Three and more	504 (49.5)	470 (56.2)	527 (31.0)	
Experience of child’s death				
No	977 (96.3)	82 (94.3)	895 (96.4)	0.303^b^
Yes	38 (3.7)	5 (5.7)	33 (3.6)	
Desire for last birth				
Want to get pregnant	843 (82.7)	74 (85.1)	769 (82.5)	0.548^b^
Not want to get pregnant	176 (17.3)	13 (14.9)	163 (17.5)	
Antenatal care provided by a medical doctor				
Yes	978 (96.0)	78 (89.7)	900 (96.6)	0.002^b^
No	41 (4.0)	9 (10.3)	32 (3.4)	
Number of antenatal care visits				
≥8 visits	482 (49.3)	34 (43.6)	448 (49.8)	0.290^b^
<8 visits	495 (50.7)	44 (56.4)	451 (50.2)	
Pregnancy gestation at the first antenatal care visit				
≤12 weeks	880 (90.9)	69 (88.5)	811 (91.1)	0.433^b^
>12 weeks	88 (9.1)	9 (11.5)	79 (8.9)	
Place of delivery				
In hospital	1,003 (98.4)	84 (96.6)	919 (98.6)	0.150^c^
Not in hospital	16 (1.6)	3 (3.4)	13 (1.4)	
Delivery assisted by a medical doctor				
Yes	909 (89.2)	79 (90.8)	830 (89.1)	0.615^b^
No	110 (10.8)	8 (9.2)	102 (10.9)	

^aFor the level of significance of 0.05 according to chi-square testb, Student’s t-testc, Fisher’s exact testd, and Mann–Whitney U-teste.^

A multivariate logistic regression model with LBW newborns as an outcome variable showed the association between women’s illiteracy (OR: 1.741; 95% CI: 1.060–2.859) and antenatal care not provided by a medical doctor (OR: 2.735; 95% CI: 1.229–6.087) (Table 2).

**Table 2:** Multivariate logistic regression with LBW newborns as an outcome variable.

Characteristics of women from low-income households	OR	95%CI OR	p*
Residence area			
Urban	1.0 reference category		0.121
Rural	0.698	0.443–1.099	
Ethnicity			
Non-Roma	1.0 reference category		0.549
Roma	1.175	0.683–1.991	
Literacy			
Yes	1.0 reference category		0.028*
No	1.741	1.060–2.859	
Age at first marriage/union			
≥18 years	1.0 reference category		0.379
<18 years	0.794	0.475–1.327	
Antenatal care provided by a medical doctor			
Yes	1.0 reference category		0.014*
No	2.735	1.229–6.087	

*p < 0.05.

## 4 Discussion

This study aimed to analyze the association between the socio-demographic and reproductive characteristics of women living in low-income households and low birth weight in Serbia, Kosovo, and Montenegro. Our results showed that illiterate women in low-income households had almost two times higher likelihood of having LBW newborns. Furthermore, women from low-income households who did not receive antenatal care from a medical doctor had a three times higher likelihood of experiencing adverse pregnancy outcomes.

The data for this study were generated using UNICEF’s methodology, which has been applied and tested in 116 countries every 5 years (57). This approach ensures a high level of data quality, coverage, comparability, and capacity for both micro and macro analyses (57). In the Western Balkans, the sampling methodology targets households in the general population as well as those in the settlements predominantly inhabited by Roma, allowing for the measurement of health indicators of women’s and children’s health in this region (46). The MICS6 methodology was applied across all three populations, including the same instruments to measure household assets and categorize them into wealth index quintiles, ensuring comparability among them. The wealth index is a composite indicator of a household’s cumulative living standards, serving as a relative measure of wealth, consumption, and income based on household assets (58). Despite its limitations—such as lack of comparability across countries and time, and differences in asset types between urban and rural areas—this indicator is widely utilized in household surveys (e.g., Demographic and Health Surveys [DHS], MICS) for developing countries, enabling consistent ranking of populations from the poorest to the richest (58, 59). Additionally, other studies have utilized the wealth index to group target populations and investigate predictors of LBW in low-resource settings (14, 60).

Numerous studies have shown that women’s illiteracy, low education levels, and poor health literacy are significantly associated with adverse pregnancy outcomes in both developing and developed countries (27, 61–64). These findings align with our results, which indicate that one-third of women residing in low-income households were illiterate. The MICS6 reports underscore the illiteracy rates among women aged 15–45 as an important indicator for women’s and children’s health (47–49). Illiteracy significantly impairs a woman’s ability to access essential information and education for maintaining or improving her health, particularly during pregnancy (27, 65). The WHO has advocated for sustainable investments in women’s education as part of the Global Strategy for Women’s, Children’s, and Adolescents’ Health (2016–2030) (66). The results of our study could catalyze the implementation of this strategy, enhancing women’s social rights and empowering their positions in the Western Balkans.

In our study, we found that low attendance at medical doctor check-ups during pregnancy among the selected sample of women from low-income households in Serbia, Kosovo, and Montenegro was a significant contributing factor to the occurrence of LBW newborns. LBW, preterm births, and intrauterine growth restrictions represent critical health challenges that require heightened attention in obstetric and neonatal care. Therefore, the WHO antenatal care guideline emphasizes the importance of maternal risk evaluation, blood analysis, and monitoring fetal growth and placental function during pregnancy (55). Effective prevention of LBW necessitates a well-trained medical team, including doctors, gynecologists, and midwives, as well as appropriate technical equipment and resources (55). Research from various regions indicates that insufficient antenatal care, characterized by a low number of visits and late engagement during pregnancy, elevates the risk of LBW (13, 67, 68). A study involving members of the European Board and College of Obstetrics and Gynecology (EBCOG) and the European Association for Perinatal Medicine analyzed the accessibility and content of antenatal care across 21 European countries (69). The findings from this study and other studies revealed that less than 10% of the analyzed countries provided specialist (gynecologist-led) care for high-risk pregnancies, including those at risk for LBW, preterm births, and intrauterine growth restriction, often relying solely on midwife services (29, 54, 69, 70). These findings, coupled with EBCOG standards, highlight the importance of doctor-led antenatal care services for effectively managing high-risk pregnancies, which aligns with our results. In Serbia, healthcare is predominantly organized around gynecologist-led care, encompassing preventive services for women of reproductive age, including family planning, antenatal care, and postnatal services at all referral healthcare levels (71). A UNICEF study on antenatal care in Kosovo found that gynecologists deliver the majority (98.5%) of these services (72). In Montenegro, the chosen gynecologist provides antenatal care on the primary level (73). Collectively, these studies demonstrate that antenatal care is primarily doctor-led across all three healthcare systems, ensuring access for all pregnant women.

The other results of our study showed that characteristics such as living in a settlement populated with Roma minority, urban residence, early marriage, and low education were significantly more present in the group of women with LBW newborns. The pattern of women’s characteristics associated with LBW newborns in our study closely resembled findings from research on LBW determinants conducted in developing and low-income countries (13, 58).

Our findings highlight socio-economic and ethnic inequalities among subpopulation groups, particularly women living in low-income areas and marginalized minority settlements. A study conducted in Hungary revealed that segregated communities, primarily inhabited by the Roma population, experience high demand for healthcare alongside elevated morbidity and mortality rates, yet receive far less funding compared to non-segregated communities (74). Previous studies conducted in Kosovo, Serbia, North Macedonia, and Hungary had also demonstrated the healthcare system’s inadequate response to the needs of Roma women (10, 50, 51, 75).

The strength of this study was the applied MICS methodology, which provided a high level of data quality and comparability among selected Western Balkans populations. The sampling methodology used in MICS enabled the investigation of characteristics of women within representative samples of households representing the general population and households in the settlements inhabited by vulnerable minority groups. The standardized MICS questionnaire used in the survey allowed for consistency in measuring the wealth index, women’s socio-economic status, and household incomes.

One of the limitations of this study is the cross-sectional design, which prevents us from examining the more specific causes and consequences of LBW in the selected Western Balkans populations of women. Another limitation is the possibility of recall bias related to the mother’s memory of the child’s birth weight data, which could question the accuracy of birthweight data in the study. This study could not address the overall multicausality of LBW related to the intrauterine restriction of fetal growth, preterm birth, or both. The MICS methodology did not account for the collection of data on other potential confounders (e.g., maternal nutrition, genetic factors), which could influence LBW. A potential limitation of this study is the use of the wealth index as a measure of income and socio-economic status of women’s households. However, this indicator of wealth is consistently used in the national population surveys.

In summary, factors associated with a low birth weight of newborns were illiteracy and antenatal care not provided by a medical doctor in the population of Serbia, Kosovo, and Montenegro. Efficient regional and national interventions are imperative to enhance women’s socio-economic status and education and to empower them to make decisions about their own health. There is an urgent need to strengthen antenatal care for the population of women in low-resource settings and harmonize the services with European standards in Western Balkans. Further studies are needed to explore the causal factors of LBW in the specific social conditions of the Western Balkans, including poverty, ethnic disparities, political and economic instabilities, and insufficient progress in welfare.

## Data Availability

Publicly available datasets were analyzed in this study. This data can be found: https://mics.unicef.org/surveys.
